# Reproductive health practices and use of health services among immigrant Indonesian women working in Malaysia

**DOI:** 10.11606/s1518-8787.2022056003811

**Published:** 2022-06-20

**Authors:** Rosnah Sutan, Pinta Pudiyanti Siregar

**Affiliations:** I University Kebangsaan Malaysia Faculty of Medicine Community Health Department Kuala Lumpur Malaysia University Kebangsaan Malaysia. Faculty of Medicine. Community Health Department. Kuala Lumpur, Malaysia

**Keywords:** Women, Transients and Migrants, Health Knowledge, Attitudes, Practice, Sexual and Reproductive Health, Health Services Accessibility

## Abstract

**OBJECTIVE:**

To describe the reproductive health practices of immigrant Indonesian women working in Malaysia and their accessibility to health services.

**METHODS:**

A cross-sectional study using a validated self-administered questionnaire was conducted with 593 immigrant Indonesian workers who stayed in Malaysia for at least six months and within the reproductive age group.

**RESULTS:**

About 13.5% of the respondents have used health facilities for reproductive health-related problems. Less than half of the respondents preferred to use public health facilities. Only 15% used treatment available in health facilities related to irregular menstrual cycles (34.6%), severe dysmenorrhea (58.7%) and nonspecific symptoms related to menstruation (31.7%). Family planning services were the most required health service. However, only 31.5% met the needs for family planning services. One-third of the respondents had sexual reproductive health problems and required treatment, but only 9.9% sought reproductive health services when needed.

**CONCLUSIONS:**

Strategies to improve the accessibility to sexual reproductive health services requires a collaboration between the Indonesian government representatives in Malaysia and non-governmental organizations to address the reproductive health issues among immigrant Indonesian women in Malaysia. Health policy related to immigrant workers is needed in order to enhance the accessibility to women’s health needs for universal health coverage.

## INTRODUCTION

Recent evidence shows inaccessibility to sexual and reproductive health (SRH) services for immigrant workers in Malaysia^1^. SRH services are best implemented across the life stages as an essential package for women, Almieda et al^4^ . Meeting the universal health coverage approach in SRH services required a good understanding of the population’s problems, including the minority and immigrant workers. Immigrant female workers have received insufficient health care and social support during the prenatal and postpartum, thus increasing the risk of postpartum depression, worsening with lack of social support^5^. Length of stay in the destination country is essential to design the health program for immigrants^6^. Evidence from literature emphasizes the barriers to health care among immigrant workers, such as language, interest in alternative treatment, unawareness, stigma and bad treatment by health care providers. Immigrants have more health problems since they need to adapt to the new environment, but they have limited access to health services. We noticed the cultural barrier as the cause of immigrants not using health services. Policy enforcement plays an important role to help immigrants overcome barriers^7^. Studies on this issue among immigrant workers are abundant, but few studies analyze the use of health services by Indonesian immigrants. Better reproductive health policy for immigrant workers is still needed in many countries, including the donor and the receiver country^8^.

Essential reproductive health services for immigrants include menstruation, menopause, contraception, screening, sexually transmitted diseases and health education^9^. However, a study in China found that immigrants were less aware of the symptoms and prevention of reproductive tract infection and lacked self-reproductive hygiene^10^. Improvement in reproductive health practice among immigrant women who received education on reproductive health may prevent health risks and improve their quality of life in the donor country^10^. Many factors may affect immigrant workers’ health. The complex procedures during the travel process may also affect their physical and mental health as well as the limited access to health care. The possibility of developing a new disease or getting the disease after immigration is another risk factor as the workers adapt to the sudden changes in situations and lifestyles^11^. We identified inaccessibility to health services and SRH services among immigrant workers^12^. However, few studies on best practices have been published to overcome the barrier.

Research comparing immigrant and native workers’ health found that immigrant workers tend to have poorer health outcomes than native workers^13^. Immigrant women have difficulty to access health services during pregnancy and childbirth, which leads to complications and endangering their lives. Local studies in Malaysia^14^reports lack of information about services and language barriers during routine health screening as issues for immigrants. The Sustainable Development Goal suggests the need to include reproductive health services into countries’ planning for immigrants to achieve universal health coverage, with family planning and education for example^16^. Therefore, this study aims to analyze Indonesian female workers’ use of health services and the reproductive health problems they face in Malaysia.

## METHODS

Three study sites in West Malaysia were chosen: (1) Kuala Lumpur’s Embassy of the Republic of Indonesia (KBRI), (2) Johor Bahru’s Consulate General of the Republic of Indonesia (KJRI Johor) and Penang’s Consulate General of the Republic of Indonesia (KJRI Penang). Permission to conduct the study was obtained from the University Kebangsaan Malaysia research and ethics committee with approved project code FF-2017-287. The study also received approval from KBRI Kuala Lumpur, KJRI Johor Bahru and KJRI Penang. The respondents were randomly selected based on a list of attendance to the stated Immigrant department (KBRI Kuala Lumpur, KJRI Johor Bahru and KJRI Penang). The respondents were informed about the study and voluntarily responded to it. Verbal and written explanations of the purpose of the study were provided to each respondent. The inclusion criteria were: (1) agreeing to take part in the study, (2) women of reproductive age (19–45 years old), (3) who had a valid permit and (4) length of stay in Malaysia of at least 12 months. We excluded Tawau’s Consulate General of the Republic of Indonesia (KJRI Tawau), Kuching’s Consulate General of the Republic of Indonesia (KJRI Kuching) and Kota Kinabalu’s Consulate General of the Republic of Indonesia (KJRI Kota Kinabalu) as these are situated in East Malaysia. The respondents who showed emotional instability, sickness and poor communication were excluded.

We measured the respondents’ background based on socioeconomic and health insurance coverage. Information on reproductive health is analyzed by a validated questionnaire about reproductive organs, menstrual cycle, vaginal discharge, family planning, sexually transmitted diseases, HIV/Aids and health rights as immigrant workers. No specific questionnaire on immigrant Indonesian workers’ SRH practices and use of SRH services is available. Therefore, we adapted the available questionnaire. The adopted questionnaire on SRH was from Cleland^17^ (1998), Symonds and Arulkumaran^18^ (2013), Hussain^19^(2017) and Azhar^20^ (2017). The expert’s reliability was reported as more than 80% agreeable for all items. The questionnaire was developed in the Indonesian language. The validation result showed the Keizer Meter-Olkin (KMO) value is 0.770, the Cronbach Alpha was 0.849, the exploratory factor analysis (EFA) test is above 0.400.

About 452 samples were part of the required minimal sample size^21^. Analysis was performed using the Statistical Package for Social Sciences (SPSS) 26.0 (Chicago, Illinois, United States). Data cleaning is conducted to detect any data loss and decode errors or any data values that are not unambiguous.

## RESULT

### Socioeconomics Distribution

A total of 593 female workers took part in this study. The mean length of stay in Malaysia was 4.1 (SD = 3.17) years and the range was between 1 and 30 years. About 80.1% of workers lived in dormitories and 89.0% were not married. The respondents are originally from Central Java (30.2%), North Sumatra (26.1%) and East Java (17.2%). Most respondents in the KJRI Penang came from North Sumatra. Probably due to the closer distance between Penang and North Sumatra and accessibility to boats or ferries to cross the border. However, many respondents at the Kuala Lumpur’s Indonesian Consulate General (KBRI) came from Java Island, which relates to the high number of Javanese living in Johor Bahru. Most respondents (95.1%) are Muslim.

The range of respondents’ age was from 19 to 45 years old and the mean age was 26.8 (SD = 6.7) years old. The largest group was less than 30 years old (76.2%). Most respondents have an education degree up to higher school (79.9%). The most popular occupation was factory workers (79.8%), followed by housemaids (7.3%), cleaning services (3.9%), restaurant workers (2.2%) and others such as field workers, information technology, administration, teachers and professionals. Most respondents work in a shifted job (77.9%). About 521 respondents (87.9%) took leave according to their needs for a few days per year or days per month. However, 72 respondents (12.1%) did not allow or know their eligibility to take leave. The working duration was between 4 and 21 hours, with a mean of 11.4 (SD = 1.8) hours a day. The study requires a working duration of about 12 hours a day (72.8%).

The respondents’ monthly income was between RM500 and RM5000. The mean income was RM1340.0 (SD = 454.0) and the median was RM1200. A total of 53% reported sending back their salaries to their family in Indonesia to support their family’s living expenses within RM200 to RM1800 (median RM600).

### Obstetric History and Use of Health Services

Most respondents are married and have children (95.7%). Only 6% of the respondents have four or more children. Respondents have given birth at least once and some up to more than four times. About 9.8% had experienced miscarriages and most of them have a nuclear family structure (80%). Only 19 respondents (17.4%) had their children cared for by extended families and babysitters.

About 442 (74.5%) respondents stated, that they have health insurance coverage and 151 (25.5%) did not have or were unaware if they have health insurance. More than half of the respondents stated that their employer paid for their health insurance. Only 18.3% (n = 109) paid health insurance by themselves. Almost half of the respondents had their treatment paid by themselves or by their employer. More than three quarters stated that they have a regular place for treatment (Table 1).

**Table 1:** Description of the respondents’ obstetric and health care information who have a regular place for treatment.

Obstetric history	n	Percentage (%)
Had obstetrics problem (n = 164)		
Never	7	4.3
One time	55	33.5
Two times	72	43.9
Three times	19	11.6
≥ Four times	11	6.7
Frequency of obstetric delivery (n = 164)		
Never	7	4.3
One time	63	38.4
Two times	69	42.1
Three times	17	10.4
≥ Four times	8	4.8
Abortion (n = 164)		
Never	148	90.2
Ever	16	9.8
Type of family structure practice in Indonesia (n = 164)		
Nuclear family	90	54.9
Extended family and others	19	11.6
Nonspecific	55	33.5
Health insurance (n = 593)		
Available	442	74.5
Not available or did not know	151	25.5
Who paid for health insurance (n = 593)		
Employer	379	63.9
Others	214	36.1
Who paid the health care fee (n = 593)		
Employer	251	42.3
Others	342	57.7
Had the usual health care (n = 593)		
Yes	452	76.2
No	141	23.8

### Reproductive Health Problems

About one-third of workers experienced irregular menstrual cycles (34.6%) and back pain without menstruating (31.7). More than half of the respondents experienced lower abdominal pain before or during menstruation (58.7%) and 47 (13.5%) used health facilities for treatment. About 15–20% use the available health facilities for medication. Less than half of the respondents have attended the nearby health facilities and used the services in the facilities. The respondents’ mainly used services for family planning (9.1%) and 31.5% had used other services (Table 2).

**Table 2:** Reproductive health problems experience.

Reproductive problems	Experience problems n (%)	Seek treatment n (%)
Lower/back abdominal pain before or during menstruation	348 (58.7)	47 (13.5)
Irregular menstrual cycle	205(34.6)	43 (21.0)
Backache without menstruation	188 (31.7)	30 (16.0)
Excessive menstruation that penetrates or clots out	110 (18.5)	18 (16.4)
Lower abdominal pain without menstruation	112 (18.9)	15 (14.2)
Lots of vaginal discharge problems, smell and itching	100 (16.9)	13 (13.0)
Urinating and clogging feeling	63 (10.6)	11 (17.5)
Itching in the pubic area	120 (20.2)	8 (6.7)
The stomach feels enlarged	80 (13.5)	7 (8.8)
Difficulty to urinate	21 (3.6)	5 (23.8)
Continuous absence of menstruation for six months or more	17 (2.9)	5 (29.4)
Bleeding from the vagina between menstrual cycles	30 (5.1)	4 (13.3)
Mental disorders (stress, anxiety and depression)	49 (8.3)	3 (6.1)
Breast problems (lumps, bleeding or pus from the nipples, uneven shape of the breasts)	12 (2.0)	1 (8.3)
Pain or bleeding during/after sex	8 (1.4)	1 (12.5)
Not pregnant after one year of marriage	5 (0.8)	0
Other symptoms	3 (0.5)	0
Health services needed		
Family planning	54 (9.1)	17 (31.5)
Routine health care examination	42 (7.1)	18 (42.9)
Pregnancy examination	22 (3.7)	6 (27.3)
HIV/Aids, hepatitis B screening	19 (3.2)	8 (42.1)
Cervix cancer screening (papanicolaou test)	18 (3.0)	7 (38.9)
Abortion	6 (1.0)	2 (33.3)
Others	3 (0.5)	2 (66.7)

### Use of Reproductive Health Services

Reproductive health problems often affect the young population. About 461 (78.3%) respondents with reproductive health problems did not seek health services. Only one-third who had the problems received treatment. About one-tenth of the respondents (n = 54, 9.1%) used reproductive health services when facing reproductive health problems (Figure).

**Figure:**
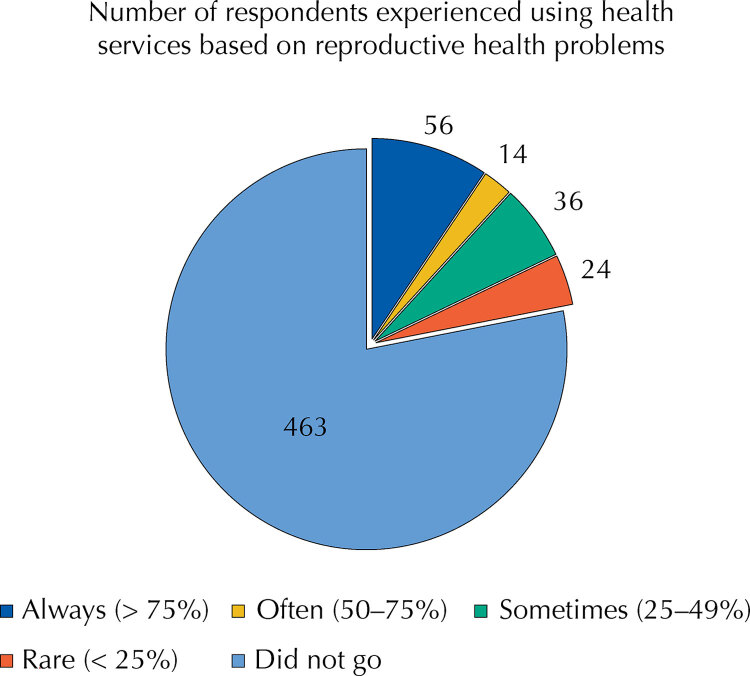
Experience using reproductive health services based on reproductive problems.

## DISCUSSION

The immigrant Indonesian women working in Malaysia were in the younger age group (mean age was 26.8 years old) compared to Thailand^22^. Most of them have not married and worked in factories, in contrast to research in Thailand that showed that most immigrants worked in restaurants and agriculture^22^. We found that immigrant Indonesian women, on average, have reproductive health problems. Only 22% of immigrant female workers used reproductive health services if they had reproductive health problems. More than half of the respondents stated that they had experienced dysmenorrhea and that this affected their daily life. A study found that immigrants require health care services due to their vulnerability to illnesses^22,23^. Recent qualitative research among stakeholders in Malaysia found that pregnant immigrant workers could not work when they were sick and needed to send them home^2^. Immigrants who take family planning are seen as practicing free sex or promiscuous^15^. We found that immigrants’ obstacles in Malaysia include financial problems, passport renewal, language barrier, discrimination, outreach problems and employers. Therefore, immigrant Indonesian workers must have health insurance coverage while staying in Malaysia that must cover primary women screening, SRH illness treatment and family planning. Not many immigrant workers had screenings. Only 3% of immigrants reported having done HIV and hepatitis B screening.

An earlier study on Chinese people who immigrated to the West had a higher prevalence of hepatitis B but delayed getting treatment and diagnosis. A systematic review found that immigrants are unaware to the access to screening and vaccines^24^. Health care providers who conduct targeted outreach screening are needed to identify those who may not seek health care services^25^. A study by a focus group discussion (FGD) shows that the obstacles for HIV testing on immigrants in Australia were cost, place to check, stigma regarding HIV and the delay in HIV diagnosis^26^. Targeted screening for HIV testing with new methods, such as rapid tests and self-testing for HIV diagnosis, can help these groups overcome barriers^26^. Immigrant women lacks understanding of reproductive health. In fact, from this study, young immigrant women are likely to become extortion victims. Their employers should emphasize the legal system’s health care service and the access for immigrant workers. However, few best practice strategies explained the successful approach to overcoming stigma and poor health care services^27,28^. All immigrants entering Malaysia are screened for pulmonary tuberculosis, following the Malaysian policy to obtain Visit Pass (Temporary Employment). Those who do not comply are sent back to their origin country and the employment certificate will not be issued by the Foreign Workers Medical Examination Monitoring Agency^35^.

One of the reasons immigrant women do not use reproductive health services is the inaccessibility to reproductive health promotion activities. More than half of Indonesian workers who immigrated to Malaysia need the services of family planning. Immigrant Indonesian workers go to work in Malaysia because of the socio-cultural and language similarities. They continue their practice in the receiver country when they immigrate. A study found that immigrants and future generations are more interested in family planning with long-term effects than native families^29^. For example, people who move either from urban to rural or rural to urban areas in Kenya use modern contraceptives, in comparison to those who do not move in their villages^30^. Evidence shows that immigrant workers experience depression and anxiety as they become victims of exploitation and work in dangerous and unsanitary places^31^. We found that 8% of the immigrant Indonesian women had mental health problems, but only 3% used health services. Those who seek health services face barriers to access the services, which leads to relapse. An earlier study by Njeru et al.^32^ found that mental illness problems can be treated and avoid relapse in most immigrants despite language and culture.

The public health sector, especially the occupational health unit, should address SRH issues among immigrant workers and include them in the health insurance coverage and promotion activities. Primary health care workers must be able to work with immigrants with SRH issues. The collaboration between government and private sectors can be a solution to promote health awareness on reproductive health services by using women-friendly health programs^33^. Health education for immigrant workers can improve immigrants’ health habits. This study suggests that online health education can help provide an overview of these immigrants^34^. Some countries implemented many solutions to increase reproductive health services for immigrants^33^. The Malaysian government has provided health facilities with essential life stages approach services for all^16^. The awareness of these facilities’ existence is unknown, especially among the immigrants who work in a factory and stay in the company’s dormitory. The foreigner usually gets low payment^35^, and most of them work in non-professional work tasks. Therefore, foreigners face a financial barrier in accessing health care facilities and services^35^.

Our study covers all Indonesian immigration departments in Malaysia, which can generalize the findings about this population. The limitation was among the illegal immigrants who were challenging to approach.

## CONCLUSION

There are SRH problems and poor health care use among immigrant Indonesian women working in Malaysia. They compose the largest group of immigrants in Malaysia. Promoting a better health habits should help improve their health and improve Malaysia’s overall health status and meet the universal health coverage target. The enforcement of employers to cover health insurance for essential SRH services emphasizes the need to recruit staff. Indonesian government representatives in Malaysia and non-governmental organizations need to address reproductive health issues among immigrant Indonesian female workers to find better strategies for reproductive health services. Health policy for immigrant workers is needed to improve women’s health needs for universal health coverage.

## References

[1] Loganathan T, Chan ZX, Smalen AW, Pocock NS. Migrant women’s access to sexual and reproductive health services in Malaysia: a qualitative study. Int J Environ Res Public Health. 2020 [cited 2021 Dec 2];17(15):5376. Available from: 10.3390/ijerph17155376 PMC7432037 32722563

[2] Loganathan T, Rui D, Ng CW, Pocock NS. Breaking down the barriers: understanding migrant workers’ access to healthcare in Malaysia. PLoS One. 2019 [cited 2021 Dec 2];14(7):e0218669. Available from: 10.1371/journal.pone.0218669 PMC6608924 31269052

[3] Lim SC, Yap YC, Barmania S, Govender V, Danhoundo G, Remme M. Priority-setting to integrate sexual and reproductive health into universal health coverage: the case of Malaysia. Sex Reprod Health Matters. 2020 [cited 2021 Dec 2];28(2):1842153. Available from: 10.1080/26410397.2020.1842153 PMC7887985 33236973

[4] Almeida, Ligia & Costa Santos, Cristina & Caldas, José & Dias, Sónia & Ayres-de-Campos, Diogo. The impact of migration on women’s mental health in the postpartum period. Rev Saude Publica. 2016 [cited 2021 Dec 2];50:1–13. Available from: 10.1590/S1518-8787.2016050005617 PMC4917335 27355463

[5] Almeida LM, Costa Santos C, Caldas JP, Dias S, Ayres-de-Campos D. The impact of migration on women’s mental health in the postpartum period. Rev Saude Publica. 2016 [cited 2021 Dec 2];50:35. Available from: 10.1590/S1518-8787.2016050005617 PMC4917335 27355463

[6] Islam MM, Gagnon AJ. Use of reproductive health care services among urban migrant women in Bangladesh. BMC Womens Health. 2016 [cited 2021 Dec 2];16(1):15. Available from: 10.1186/s12905-016-0296-4 PMC4785632 26961123

[7] Satinsky E, Fuhr DC, Woodward A, Sondorp E, Roberts B. Mental health care utilization and access among refugees and asylum seekers in Europe: a systematic review. Health Policy. 2019 [cited 2021 Dec 2];123(9):851-63. Available from: 10.1016/j.healthpol.2019.02.007 30850148

[8] Endler M, Al Haidari T, Chowdhury S, Christilaw J, El Kak F, Galimberti D, et al. Sexual and reproductive health and rights of refugee and migrant women: gynecologist’s and obstetricians’ responsibilities. Int J Gynecol Obstet. 2020 [cited 2021 Dec 2];149(1):113-9. Available from: 10.1002/ijgo.13111 32012258

[9] Ussher JM, Perz J, Metusela C, Hawkey AJ, Morrow M, Narchal R, et al. Negotiating discourses of shame, secrecy, and silence: migrant and refugee women’s experiences of sexual embodiment. Arch Sex Behav. 2017 [cited 2021 Dec 2];46(7):1901-21. Available from: 10.1007/s10508-016-0898-9 PMC5547186 28083724

[10] Xu S, Yu C, Zhou Y, Wu J, Bai T, Zhang J, et al. The prevalence of reproductive tract infections in a Chinese internal migrant population, and its correlation with knowledge, attitude, and practices: a cross-sectional study. Int J Environ Res Public Health. 2019 [cited 2021 Dec 2];16(4):655. Available from: 10.3390/ijerph16040655 PMC6406905 30813340

[11] Hargreaves S, Rustage K, Nellums LB, McAlpine A, Pocock N, Devakumar D, et al. Occupational health outcomes among international migrant workers: a systematic review and meta-analysis. Lancet Glob Health. 2019 [cited 2021 Dec 2];7(7):e872-82. Available from: 10.1016/S2214-109X(19)30204-9 PMC6565984 31122905

[12] Simkhada P, Teijlingen E, Gurung M, Wasti SP. A survey of health problems of Nepalese female migrants’ workers in the Middle East and Malaysia. BMC Int Health Hum Rights. 2018 [cited 2021 Dec 2];18(1):4. Available from: 10.1186/s12914-018-0145-7 PMC5774120 29347938

[13] Pham KTH, Nguyen LH, Vuong QH, Ho MT, Vuong TT, Nguyen HKT, et al. Health inequality between migrant and non-migrant workers in an industrial zone of Vietnam. Int J Environ Res Public Health. 2019 [cited 2021 Dec 2];16(9):1502. Available from: 10.3390/ijerph16091502 PMC6539052 31035337

[14] Sami J, Lötscher KCQ, Eperon I, Gonik L, Martinez de Tejada B, Schmidt NC. Giving birth in Switzerland: a qualitative study exploring migrant women’s experiences during pregnancy and childbirth in Geneva and Zurich using focus groups. Reprod Health. 2019 [cited 2021 Dec 2];16(1):112. Available from: 10.1186/s12978-019-0771-0 PMC6647303 31331344

[15] Kamaluddin SF. A follow-up profile of women seeking pregnancy terminations in a clinic in urban Malaysia: 1998-2005. Jurnal Sains Kesihatan Malaysia. 2010 [cited 2021 Dec 2];8(1):5-11. Available from: http://journalarticle.ukm.my/3658/1/A_Follow-up_Profile_of_Women_Seeking_Pregnancy_Terminations.pdf

[16] Ab Rahman N, Sivasampu S, Noh KM, Khoo EM. Health profiles of foreigners attending primary care clinics in Malaysia. BMC Health Serv Res. 2016 [cited 2021 Dec 2];16:197. Available from: 10.1186/s12913-016-1444-0 PMC4908717 27301972

[17] Cleland J. Illustrative questionnaire for interview with young people. Geneva (CH): WHO; 1998 [cited 2021 Dec 2]. Available from: http://www.who.int/reproductivehealth/topics/adolescence/questionnaire.pdf

[18] Symonds I, Arulkumaran SS. Essential Obstetric and Gynaecology. 5. ed. London (UK): Churchill Livingstone; 2013.

[19] Hussain NHN. Ginekologi Ilmu Kesihatan Wanita. Kuala Lumpur (MY): Dewan Bahasa dan Pustaka; 2017.

[20] Azhar MN. Simplified Gynaecology for medical students. Shah Alam (MY): Reka Cetak Sdn Bhd; 2017.

[21] Tamil AM. How to calculate your own sample size. Kuala Lumpur (MY): Secretariat of Medical Centre & Industry, UKM Medical Centre; 2008 [cited 2021 Dec 2]. Available from: https://issuu.com/azmimohdtamil/docs/howtocalculateyourownsamplesize2008

[22] Khongthanachayopit S, Laohasiriwong W. Accessibility to health services among migrant workers in the Northeast of Thailand. F1000Res. 2017 [cited 2021 Dec 2];6:972. Available from: 10.12688/f1000research.11651.1 PMC5615766 29034076

[23] Shao S, Wang M, Jin G, Zhao Y, Lu X, Du J. Analysis of health service utilization of migrants in Beijing using Anderson health service utilization model. BMC Health Serv Res. 2018 [cited 2021 Dec 2];18(1):462. Available from: 10.1186/s12913-018-3271-y PMC6006712 29914464

[24] Vedio A, Liu EZH, Lee ACK, Salway S. Improving access to health care for chronic hepatitis B among migrant Chinese populations: a systematic mixed-methods review of barriers and enablers. J Viral Hepat. 2017 [cited 2021 Dec 2];24(7):526-40. Available from: 10.1111/jvh.12673 PMC5516707 28092419

[25] Gray C, Lobo R, Narciso L, Oudih E, Gunaratnam P, Thorpe R, et al. Why I can’t, won’t or don’t test for HIV: insights from Australian migrants born in sub-Saharan Africa, Southeast Asia and Northeast Asia. Int J Environ Res Public Health. 2019 [cited 2021 Dec 2];16(6):1034. Available from: 10.3390/ijerph16061034 PMC6466030 30901957

[26] Ivanova O, Rai M, Kemigisha E. A systematic review of sexual and reproductive health knowledge, experiences and access to services among refugee, migrant and displaced girls and young women in Africa. Int J Environ Res Public Health. 2018 [cited 2021 Dec 2];15(8):1583. Available from: 10.3390/ijerph15081583 PMC6121882 30049940

[27] Vissandjée B, Short WE, Bates K. Health and legal literacy for migrants: twinned strands woven in the cloth of social justice and the human right to health care. BMC Int Health Hum Rights. 2017 [cited 2021 Dec 2];17(1):10. Available from: 10.1186/s12914-017-0117-3 PMC5390456 28403844

[28] Rachmawati I, Dewi MA. Model of integrative border diplomacy in managing harmony between Indonesia and Malaysia: a case of Temajuk, West Kalimantan, Indonesia. Geogr Malaysian J Soc Space. 2021 [cited 2021 Dec 2];17(1):44-56. Available from: 10.17576/geo-2021-1701-04

[29] Noor NM, Kahalid JR, Muzafar PMM. Social inequalities and health in Malaysia: The state of households 2020 - Part III. Kuala Lumpur (MY): Khazanah Research Institute; 2020 [cited 2021 Dec 2]. Available from: http://www.krinstitute.org/Publications-@-Social_Inequalities_and_Health_in_Malaysia.aspx

[30] Ochako R, Askew I, Okal J, Oucho J, Temmerman M. Modern contraceptive use among migrant and non-migrant women in Kenya. Reprod Health. 2016 [cited 2021 Dec 2];13(1):67. Available from: 10.1186/s12978-016-0183-3 PMC4888625 27246329

[31] Mucci N, Traversini V, Giorgi G, Tommasi E, De Sio S, Arcangeli G. Migrant workers and psychological health: a systematic review. Sustainability. 2020 [cited 2021 Dec 2];12(1):120. Available from: 10.3390/SU12010120

[32] Njeru JW, DeJesus RS, St Sauver J, Rutten LJ, Jacobson DJ, Wilson P, et al. The utilization of a mental health collaborative care model among patients who require interpreter services. Int J Mental Health Syst. 2016 [cited 2021 Dec 2];10:15. Available from: 10.1186/s13033-016-0044-z PMC4772682 26933447

[33] Mengesha ZB, Perz J, Dune T, Ussher J. Preparedness of health care professionals for delivering sexual and reproductive health care to refugee and migrant women: a mixed methods study. Int J Environ Res Public Health. 2018 [cited 2021 Dec 2];15(1):174. Available from: 10.3390/ijerph15010174 PMC5800273 29361799

[34] Li X, Yang H, Wang H, Liu X. Effect of health education on healthcare-seeking behaviour of migrant workers in China. Int J Environ Res Public Health. 2020 [cited 2021 Dec 2];17(7):2344. Available from: 10.3390/ijerph17072344 PMC7177837 32235675

[35] Noor NM, Khalidi JR, Muzafar PMM. Social inequalities and health in Malaysia: the state of households 2020; Part III. Kuala Lumpur (MY): Khazanah Research Institute; 2020 [cited 2021 Dec 2]. Available from: http://www.krinstitute.org/Publications-@-Social_Inequalities_and_Health_in_Malaysia.aspx

